# Institutional investment in addictive industries: an important commercial determinant of health

**DOI:** 10.3389/fpubh.2024.1409648

**Published:** 2024-10-14

**Authors:** Sébastien Berret, Virve Marionneau, Riikka Sievänen, Janne Nikkinen

**Affiliations:** ^1^Centre for Research on Addiction, Control, and Governance, Faculty of Social Sciences, University of Helsinki, Helsinki, Finland; ^2^KPMG Finland, Helsinki, Finland

**Keywords:** commercial determinants of health, responsible investment, public health, tobacco, alcohol, gambling, cannabis

## Abstract

**Background:**

The production of addictive commodities is subject to a range of commercial determinants. There has nevertheless been a gap in understanding how investments into addictive commodities may function as commercial determinants. Institutional investors can yield important financial power with their investment decisions. Many investors apply responsible investment (RI) policies to address environmental, social, and governance concerns. Negative screening is used to exclude certain sectors or companies from investment portfolios, mainly for ethical concerns. Negative screening also affects investment into tobacco and other addictive industries. This article investigates RI policies toward addictive industries among institutional investors that are signatories of the Tobacco-Free Finance Pledge (TFFP; *N* = 161). The TFFP is an initiative created in 2021 to de-normalize tobacco-related investments.

**Methods:**

The mixed-method study uses descriptive statistics to quantify the extent and scope of exclusion policies as well as institutional and geographical profiles of investors, and a qualitative analysis of the justifications for these exclusion policies.

**Results:**

Some TFFP signatories apply negative screening to other addictive industries (gambling: 35%; alcohol: 24%; cannabis 12% of signatories). There are important differences in the applied exclusion thresholds, with only 47% of TFFP signatories applying a zero-tolerance policy to tobacco. Thresholds are higher for other addictive industries. Signatories also differ in terms of their geographical and investor profiles. Justifications pertaining to compliance with international standards and reputational risks were the most common.

**Conclusion:**

Addictive industries, such as tobacco, alcohol, gambling, and recreational cannabis, are increasingly excluded by investors. However, different understandings of RI influence how sector exclusions are implemented. Divesting from tobacco and other addictive industries is a crucial step toward a public health approach that prioritizes population health over financial profits. Prominent institutional investors are influential opinion leaders who can change the behavior of other investors and de-normalize controversial industries and reduce or prevent harm.

## 1 Introduction

Tobacco, alcohol, recreational cannabis, and gambling are major public health issues with global ramifications (1–3). ‘Non-communicable’ diseases resulting from lifestyle choices such as diet, smoking, or alcohol use cause 71 percent of global deaths (4). Gambling is similarly connected to severe harms, including suicidality and high rates of indebtedness (75–77). The production of these health-harming products is entangled with a range of commercial determinants of health. Commercial determinants of health pertain to commercial companies’ activities that market goods or services with the potential to harm health (5). Commercial determinants include unhealthy commodities, business and political practices to sell these commodities, and global drivers that impede regulation (78).

Increasing research attention is directed to the commercial determinants of tobacco and other addictive industries (5–9, 79). Addictive industries consist of companies involved in the provision of addictive or risk commodities that manufacture, market, and distribute harmful products globally and drive risky consumption (10, 11). The sectors examined in this study, that is, tobacco, alcohol, recreational cannabis, and gambling, will be referred to as “addictive industries.” Other scholars have used terms such as “addiction industries” (12) or “sin stocks” (13) to describe them. These industries share common characteristics, such as their addictive nature, legality, and controversial aspects.

Research has shown that addictive industry actors maintain and create new markets by engaging in extensive marketing and lobbying (14–16), by promoting industry self-regulation via corporate social responsibility (CSR) policies including Codes of Conduct (17, 18), and by increasing their global importance via ‘corporate citizenship’ and extensive supply chains (5, 6, 19). However, there has been an important gap in our understanding of how investors and investment decisions can function as a commercial determinant, particularly within the field of addictive commodities.

Financial investors are key actors within commercial systems (20). Particularly the investment decisions of institutional investors yield important power within markets due to their size, access to policy makers, and long-term investment horizon (21). Institutional investors are companies, such as banks or pension funds, that invest money on behalf of their clients or members. Investors can exclude certain industries because of voluntary ethical commitment or in reaction to public health campaigns that are likely to damage their public image. The principle of responsible investment (RI) has become increasingly popular and even mainstream following the World Economic Forum report in 2005 and the launch of the United Nations Principles for Responsible Investment (UNPRI) in 2006 (21). Institutional investors now apply environmental, social, and governance (ESG) factors to their investment decisions [e.g., (22)]. The most common RI practice consists of negative screening, i.e., the exclusion of sectors and companies that contradict with the investor’s values (23). Exclusion lists do not make a difference between companies operating in certain sectors and therefore provide a simple tool for investors (24, 25).

The main aims of RI and negative screening are to help investors balance moral considerations and the optimization of financial risk return (26, 27). Literature has been inconclusive regarding the financial outcomes of RI. Some studies have suggested that strict RI policies may lower returns and result in financial underperformance [e.g., (28)]. Others suggest that RI portfolios can yield similar returns to portfolios without exclusion policies (22, 29–33) or even add value or increase financial performance (34–37). It is likely that performance is impacted by methodological choices (32, 38, 39). Prior research into RI policies has shown important heterogeneity in terms of adoption and expectations, as well as different levels of negative screening, exclusion thresholds, and discrepancies in the treatment of different products [e.g., (27, 39, 40)].

The prominent role of institutional investors in the global economy, and their choice to either invest in or divest from certain sectors, can have important implications on public health (41, 42). Investment decisions are likely to be a significant commercial determinant of health in the field of tobacco and other addictive commodities. Investors can choose to allocate capital to operators involved in addictive sectors and contribute to their global growth. In contrast, investors can also exert pressure on corporate behavior (21). Excluding harmful sectors from investment portfolios can limit the growth of these sectors, and the harm they generate. In this light, responsible investment policies can contribute to reducing the harm caused by addictive industries. Yet, surprisingly little research has gone into investigating institutional investment in the field of addictive commodities. One survey of US-based RI mutual funds (43) found that, while 98 percent of survey respondents excluded tobacco, only 80 percent excluded alcohol and 79 percent excluded gambling (43). Another survey study by Morgan Stanley (44) similarly found that exclusion of tobacco was more common than exclusion of gambling or alcohol. Exclusion policies were more prevalent in Europe than in North America or in the Asia-Pacific region.

The need to exclude addictive industries from investment portfolios is increasingly discussed in RI policies. A significant step forward in reducing investment in these industries was the so-called Tobacco-Free Finance Pledge (TFFP), developed at the initiative of the United Nations Environment Program in 2021. The signatories of the pledge agree to renounce investment in tobacco, primarily because of the commercial determinants of harm associated with this industry. The TFFP also aims at raising awareness about the harms caused by the tobacco industry and de-normalizing financial and corporate associations with tobacco companies (45). There are currently no similar pledges in place for other addictive industries.

The current paper presents a statistical and qualitative analysis of the exclusion policies of institutional investors toward four addictive industries: tobacco, alcohol, gambling, and recreational cannabis, among signatories of the TFFP (*N* = 161). Recreational cannabis was included in this study, as it is legal in a growing number of jurisdictions across the world. While the authors acknowledge the increase of Internet gaming disorder and the growing convergence between gaming and gambling (46), the gaming industry was not included in this study. The aim is to study whether signatories extend negative screening practices to other addictive industries, what kind of the exclusion thresholds they apply to tobacco and other sectors, variations in size, geographical location, and investor profiles of TFFP signatories, and justifications behind exclusion policies. In the following, we first describe the data and methods and proceed to present the results. Findings are discussed in terms of their public health and financial implications.

To the authors’ knowledge, this study is one of the first to examine the commercial determinants of health by focusing on the role of institutional investors in allocating capital to industries whose products are detrimental to health. This paper fits into recent collective efforts to comprehensively report on the complex aspects through which corporate influence can be exerted and its impact on public health (47), and aims to better conceptualize the multifaceted challenges posed by addictive industries. Analyzing how their exclusion from investment portfolios is justified is paramount to understanding what can be done to address the harm they cause. The present study is a first step in improving our knowledge of institutional investors’ attitudes toward RI policies excluding addictive industries and can be used to expand further research on the public health consequences of investing in and divesting from such sectors.

## 2 Methods

### 2.1 Data

Our cross-sectional dataset consists of UNPRI transparency reports of signatories of the Tobacco-Free Finance Pledge in 2020 (*N* = 161). These were the latest data available at the time of the data collection. Although the TFFP was signed in 2021, investors had already excluded the tobacco sector prior to this. We were not able to produce a longitudinal analysis as UNPRI reporting was changed in 2023, affecting comparability. The PRI has made significant modifications to the questions and indicators due to system errors in the 2021 reporting cycle (48).

UNPRI transparency reports are an output of the UNPRI Reporting Framework. Their primary objective is described as ‘*enabling signatory transparency on responsible investment activities and facilitate dialogue between investors and their clients, beneficiaries, and other stakeholders’* (UNPRI reporting Framework, 2020). UNPRI transparency reports are publicly available and provide information on the responsible investment practices of signatories, including exclusion lists or policies.

The data collection process was as follows. The 2020 UNPRI report of every TFFP signatory that signed the pledge during the data collection was examined by SB and VM. When a UNPRI transparency report was not available or if data on exclusion thresholds were missing, we searched signatories’ RI reports on their websites for this information. We found publicly available exclusion policies of addictive industries for 71 percent of the TFFP signatories. Drawing on this sample, we then identified relevant sections in the reports concerning addictive industries. The keywords used to conduct the searches were the following: exclusion policy, exclusionary screen, screening, responsible investment, avoidance list, divestment list, restricted securities list, and sustainability policy. The keywords were derived from empirical evidence, as investors use a large range of terms to refer to RI and exclusion policies.

In cases where no information was provided, the investors’ exclusions were categorized as ‘unknown’. We systematically examined the UNPRI reports of the TFFP signatories to garner data on each signatory’s exclusion policy regarding tobacco, alcohol, gambling, and cannabis. Some signatories only published reports on their exclusion of controversial weapons or fossil fuels – and not on other sectors. Approximately one-third of the signatories did not disclose information about their exclusion threshold for tobacco (35 percent not reported), alcohol (31 percent), gambling (34 percent), and cannabis (27 percent).

### 2.2 Statistical methods

Results are reported using descriptive statistics due to the relatively small number of signatories. Descriptive statistics allow describing how institutional investors implement negative screening and summarizing the data to identify key variables. We quantified the extent and scope of exclusion policies as well as institutional and geographical profiles of investors.

The descriptive categories fall under three variables: assets under management (AUM, in US dollars), investor type, and legal (geographical) origin.

AUM refers to the total value of financial assets that a financial firm manages on behalf of its clients (e.g., stocks, bonds, real estate, or other types of investments). AUM is commonly used as a measure of the size and scale of an institutional investor. The diversity of AUM among the TFFP signatories is considerable, ranging from $40 million to $1 trillion. The AUM variable was divided into seven categories of equivalent size. The largest group consisted of investors with over $100,000 million in total assets and the smallest group of investors with $0–999 million in total assets. A large proportion of investors did not provide any information on their AUM (43 percent).

The investor type comprises six categories following Ryan and Schneider (49): asset managers, banks, corporate pension funds, non-corporate pension funds, insurance companies, and other asset owners. The first aggregates various asset, fund, and investment managers across asset classes as well as related advisory. Other asset owners include reserve–sovereign or government-controlled funds, endowment funds, and foundations. The five foundations included in the sample were added to the other asset owner’s category, which includes university endowment funds and reserve–sovereign or government-controlled funds.

The legal origin category was drafted based on the location of investor headquarters. The Philippines and Bangladesh are categorized as ‘Asia’. ‘Europe’ includes European countries and the British Cayman Islands. ‘North America’ includes the United States, Canada, and Mexico. ‘Oceania’ regroups Australia and New Zealand.

### 2.3 Qualitative methods

We also conducted a qualitative analysis of the justifications used in the UNPRI reports pertaining to the sector or product exclusions. SB examined the UNPRI Transparency Report for each TFFP signatory and systematically coded when an exclusion policy was mentioned and justified with a rationale for exclusion. Coding was double-checked by VM, and a dual-coder agreement was reached. A total of 682 quotations (i.e., statements about exclusion policies accompanied with a justification) from 87 UNPRI reports were coded, with an average of seven quotations per report. The lowest number of quotations was two and the highest 18, which can be explained by the different lengths of investors’ UNPRI report (see Table 1).

**Table 1:** Descriptive statistics of the quotations count.

Count	Sum	Mean	Median	Mode	SD	Min	Max	Confidence level (95%)
87	682	7,84	7	5	3,75	2	18	0,80

The rationales used to justify sector exclusion gives insight into institutional investors’ perspective on RI but also on their moral stand in public. We therefore employed a theory-driven approach to analyze these. The analysis followed the justification analysis (JA) framework (50), which allowed us to illustrate the moral precepts behind the investors’ exclusion policies.

JA builds on the sociological theory of Luc Boltanski and Laurent Thévenot on moral justifications (1991). Boltanski and Thévenot posit that in a non-violent conflict situation, social actors possess moral capacities that enable them to ‘make society’ based on an ability to justify one’s claims (51, 52). The theory focuses on the various justifications that social actors may draw upon to advance their interests or to defend themselves during public disputes. These public claims are made in the name of the common good and hinge on different philosophical grounds with regard to moral worth (50). The JA framework therefore underscores the moral components, values, and principles that guide different types of actors making public claims (50).

The UNPRI transparency reports provided by most signatories of the TFFP include different types of justifications on their RI approach and exclusion policy. These justifications are used to convey conceptions of responsible investment or to engage in public disputes (e.g., to prevent reputational risks). We included only statements or claims that were accompanied by an argument about RI general approach or exclusion policy, as the JA approach considers explicit public claims and excludes statements that are only descriptive.

Statements explaining why the signatory implemented the RI policy or exclusion policy were systematically coded using the six categories of analysis provided by the JA framework. For example, when a TFFP signatory adopted an exclusion policy because including environment, social, and governance (ESG) criteria can have a material impact on short-term financial performance, this statement is a public claim about the investor’s RI strategy that is coupled with a justification for the claim. Such a claim can be found, among others, in the UNPRI report of signatory n.2 (2020, p. 91), and is justified by market worth due to the focus of the rationale on financial returns (see below for more information on the different categories of justifications).

Statements that simply affirmed the RI policy or sector exclusion without providing a rationale for the claim were not included in the analysis. The justifications used in UNPRI reports tended to address the signatories’ approach to RI in general, and specific product or sector exclusions were not necessarily accompanied by an argument. Therefore, it would have been too restrictive to focus only on claims about reasons for excluding the addictive products sector.

Each available UNPRI report from 2020 was imported to the qualitative data software Atlas.ti. We conducted a preliminary holistic coding based on the material. Subsequently, we performed a second round of coding to merge or modify nodes into categorizations that followed the JA framework. The JA framework has six predefined categories (50) that correspond to the six ‘orders of worth’ on which public justifications are built in Boltanski and Thèvenot’s theory:

1. The inspired worth focuses on inspiration, creativity, spirituality, or religious devotion.
2. The domestic worth is premised on tradition and hierarchy.
3. The worth of fame is defined by the predominance of public opinion, influence, and reputation.
4. The civic worth is based on the collective will, equality, solidarity, and representativeness.
5. The market worth is rooted in competition, self-interest, and money.
6. The industrial worth emphasizes efficiency, science, technology, and performance.

## 3 Results

Table 2 introduces key characteristics of the data in terms of legal origin, investor type, AUM, and sector exclusions. Overall, 35 percent of the TFFP signatories excluded gambling (35 percent), followed by alcohol (24 percent). Only 12 percent excluded cannabis.

**Table 2:** Characteristics of the tobacco-free finance pledge signatories.

Category	Number of investors	Percentage of investors
Legal origin		
Europe	86	53%
North America	20	12%
Oceania	52	32%
Asia	3	2%
Unknown	0	0%
Total	161	100%
Investor type		
Bank	13	8%
Insurance	12	7%
Asset manager	49	30%
Corporate pension fund	11	7%
Non-corporate pension fund	26	16%
Other asset owner	17	11%
Unknown	33	21%
Total	161	100%
Assets under management (M€)		
€0–999	9	6%
€1,000–4,999	14	9%
€5,000–9,999	10	6%
€10,000–24,999	10	6%
€25,000–49,999	16	10%
€50,000–99,999	14	9%
€100,000–	18	11%
Unknown	70	43%
Total	161	100%
Sector exclusion (tobacco excluded)		
Alcohol	39	24%
Gambling	57	35%
Cannabis	19	12%
No information provided on one sector or more	46	29%

In the following, we analyze the selected institutional characteristics that may impact exclusion policies in addictive industries in terms of exclusion thresholds, legal origin, and investor type, as well as the justifications employed in exclusion policies.

### 3.1 Exclusion thresholds

All TFFP signatories exclude tobacco, and an important share of the signatories also exclude other addictive industries. However, there is important heterogeneity in terms of their exclusion thresholds, or the inner scope of exclusions. The exclusion threshold shows the acceptable percentage of turnover of a company from the addictive industry for an investor. The results presented in Table 3 show that 47 percent of the TFFP signatories have a zero-tolerance policy toward tobacco. At the same time, 18 percent of the signatories invest in companies deriving 5 % or more of their revenue from tobacco production. Three signatories have an exclusion threshold set above 20 percent of revenue.

**Table 3:** Exclusion threshold of revenue derived from production or manufacture for addictive industries among the tobacco-free finance pledge signatories.

	Tobacco	Alcohol	Gambling	Cannabis
Zero-tolerance policy	75 (47%)	11 (7%)	11 (7%)	9 (6%)
5 percent threshold	16 (10%)	14 (9%)	23 (14%)	7 (4%)
10 percent threshold	8 (5%)	4 (2%)	8 (5%)	0 (0%)
20 percent threshold	2 (1%)	0 (0%)	1 (1%)	0 (0%)
Threshold over 20 percent	3 (2%)	0 (0%)	0 (0%)	0 (0%)
No threshold	0* (0%)	82 (51%)	63 (39%)	101 (63%)
Unknown	57 (35%)	50 (31%)	55 (34%)	44 (27%)
Total	161	161	161	161

*All Tobacco-Free Finance Pledge signatories providing information on their exposure to tobacco have a threshold of exclusion.

Most signatories did not have exclusion thresholds toward other addictive industries—meaning that they invest in these industries. 6–7% of those who did have an exclusion policy, applied a zero tolerance to alcohol, gambling, and cannabis. A 5-percent threshold was more common for alcohol and gambling investment. Out of the 43 investors that reported a threshold for gambling, 23 have set it at 5 %. Investors that had a zero-tolerance or low-tolerance level policy consisted particularly of European investors and investment funds.

These exclusion thresholds only include exclusions related to the production of addictive commodities. Investors may also have different thresholds for excluding, for example, retail or other related services such as marketing. While these were not possible to systematically map, a qualitative reading of the RI reports suggests that some signatories of the TTFP exclude only companies involved in tobacco production, while others also include retail or any businesses that even indirectly benefit from tobacco.

### 3.2 Legal origin

Table 4 summarizes findings related to the legal origin of the TFFP signatories. Results show that European investors make up just over half of all signatories of the pledge (53 percent) and are also highly represented in the exclusion of other addictive industries. Investors from Oceania, and notably Australia, are also well represented. The three Asian signatories in the sample did not provide any information on their sector exclusion policies. None of the TFFP signatories are from Africa or South America.

**Table 4:** Sector exclusion by legal origin among the tobacco-free finance pledge signatories.

	Tobacco	Alcohol	Gambling	Cannabis
Europe	86 (100%)	28 (32%)	38 (44%)	16 (19%)
North America	20 (100%)	4 (20%)	5 (25%)	1 (5%)
Oceania	52 (100%)	7 (13%)	14 (27%)	2 (4%)
Asia	3 (100%)	0 (0%)	0 (0%)	0 (0%)
Total	161 (100%)	39 (24%)	57 (35%)	19 (12%)

Percentages do not add up to 100, as investors can have an exclusion policy for each industry sector and as not all investors have an exclusion policy for each of the addictive sectors.

The exclusion of gambling is more commonplace among the signatories than the exclusion of alcohol or cannabis. Almost half (44 percent) of the European institutional investors exclude gambling, while about one-fourth (25 percent and 27 percent, respectively) of North American and Oceania-based investors screen out this industry. These results align with the Morgan Stanley survey (44), according to which the exclusion of gambling was also higher in Europe (32 percent of respondents) than in Asia Pacific or North America (19 percent and 14 percent, respectively). The exclusion of recreational cannabis is limited in the sample.

A closer look at the European TFFP signatories shows some disparities (Table 5). Western European investors constitute almost half of all signatories (46 percent). However, even though Western Europe is highly represented among the signatories, Central and Northern European signatories exclude alcohol, gambling, and cannabis to a larger extent than Western European or United Kingdom (UK)-based signatories.

**Table 5:** Legal origin of European tobacco-free finance pledge signatories and sector exclusions of addictive industries.

	Tobacco	Alcohol	Gambling	Cannabis
United Kingdom	12 (100%)	2 (17%)	2 (17%)	0 (0%)
Central Europe	11 (100%)	5 (45%)	7 (64%)	0 (0%)
Western Europe	40 (100%)	9 (23%)	14 (35%)	2 (5%)
Northern Europe	22 (100%)	11 (50%)	14 (64%)	14 (64%)
Eastern Europe	1 (100%)	1 (100%)	1 (100%)	0 (0%)
Total N (%)	86 (100%)	28 (33%)	38 (44%)	16 (19%)

United Kingdom includes Jersey and British Cayman Islands; Central Europe includes Germany, Luxembourg, and Switzerland; Western Europe includes France, the Netherlands, Belgium, and Spain; Northern Europe includes Sweden, Finland, Denmark, and Norway; Eastern Europe includes Estonia. N.B., investors from some large European countries, such as Italy, are not represented among the signatories.

The exclusion of alcohol and gambling is more represented among investors from Northern Europe (50 percent and 64 percent, respectively) or Central Europe (45 percent and 64 percent, respectively) than among investors from other parts of the continent. The exclusion of recreational cannabis is much higher among Northern European investors (64 percent) than other European investors. Only 5 % of Western European investors exclude cannabis, while no signatories from other European regions exclude it. The extent of exclusion is lower in the UK and Western Europe than in the Northern and Central parts of Europe. Only 17 percent of UK-based signatories screen out both alcohol and gambling, while among other Western European investors, 23 percent exclude alcohol and 35 screen out gambling.

### 3.3 Investor types

Sector exclusions by investor categories among the Tobacco-Free Finance Pledge signatories are described in Table 6. The results show that 128 investors (79 percent) disclosed their investor category. Asset managers (30 percent of all investors) are the largest group, and asset managers exclude alcohol, gambling, and cannabis to a higher degree than other investor types. We find that European investors are highly represented among asset managers (75 percent of investors). This clustering between legal origin and investor type partly explains why the asset management category appears to screen out addictive industries to a higher degree than other types of institutional investors among the signatories of the pledge.

**Table 6:** Sector exclusion by investor type among the tobacco-free finance pledge signatories.

	Tobacco	Alcohol	Gambling	Cannabis
Bank	13 (100%)	4 (31%)	4 (31%)	1 (8%)
Insurance	12 (100%)	1 (8%)	4 (33%)	1 (8%)
Asset manager	49 (100%)	21 (43%)	30 (61%)	11 (22%)
Corporate pension fund	11 (100%)	2 (18%)	3 (27%)	1 (9%)
Non-corporate pension fund	26 (100%)	4 (15%)	5 (19%)	1 (4%)
Other asset owner	17 (100%)	2 (12%)	5 (9%)	3 (8%)
Unknown	33 (100%)	5 (15%)	6 (18%)	1 (3%)
Total N (%)	161 (100%)	39 (24%)	57 (35%)	19 (12%)

### 3.4 Assets under management

Table 7 details the sector exclusion of the TFFP signatories by portfolio size. Data on the portfolio size were found for only 91 signatories (57 percent of the sample). The results suggest that investors of different sizes vary in terms of their approach to sector exclusion of addictive industries.

**Table 7:** Sector inclusion by portfolio size among the tobacco-free finance pledge signatories (percentage of category in brackets).

Size in M$	Tobacco	Alcohol	Gambling	Cannabis
Under USD 999	9 (100%)	5 (55%)	6 (67%)	1 (11%)
USD 1,000–4,999	14 (100%)	8 (57%)	11 (79%)	3 (21%)
USD 5,000–9,999	10 (100%)	3 (30%)	4 (40%)	3 (30%)
USD 10,000–24,999	10 (100%)	3 (30%)	3 (30%)	1 (10%)
USD 25,000–49,999	16 (100%)	1 (6%)	3 (19%)	3 (19%)
USD 50,000–99,999	14 (100%)	3 (21%)	5 (36%)	3 (21%)
Over USD 100,000	18 (100%)	7 (39%)	10 (56%)	3 (17%)
Unknown	70 (100%)	9 (13%)	15 (21%)	2 (3%)
Total N (%)	161 (100%)	39 (24%)	57 (35%)	19 (12%)

Only 91 investors had publicly accessible information on their AUM in their RI transparency reports of 2020.

The exclusion of alcohol and gambling is somewhat polarized. The highest numbers of exclusions were found among signatories with the lowest (under USD 999 million and between USD 1,000–4,999 million) and the largest portfolio sizes (over USD 100,000 million). Mid-range portfolio sizes were connected to comparatively low rates of exclusions. Particularly the category consisting of investors with a portfolio between USD 25,000 million and USD 49,999 million is underrepresented in terms of exclusions of other addictive industries than tobacco. Part of this result may be attributable to the fact that European investors are typically among those with the largest investment portfolios. In terms of cannabis exclusion, the low numbers of total excluders are reflected in the results as a more even distribution.

### 3.5 The justifications for the RI approach

**Figure 1:**
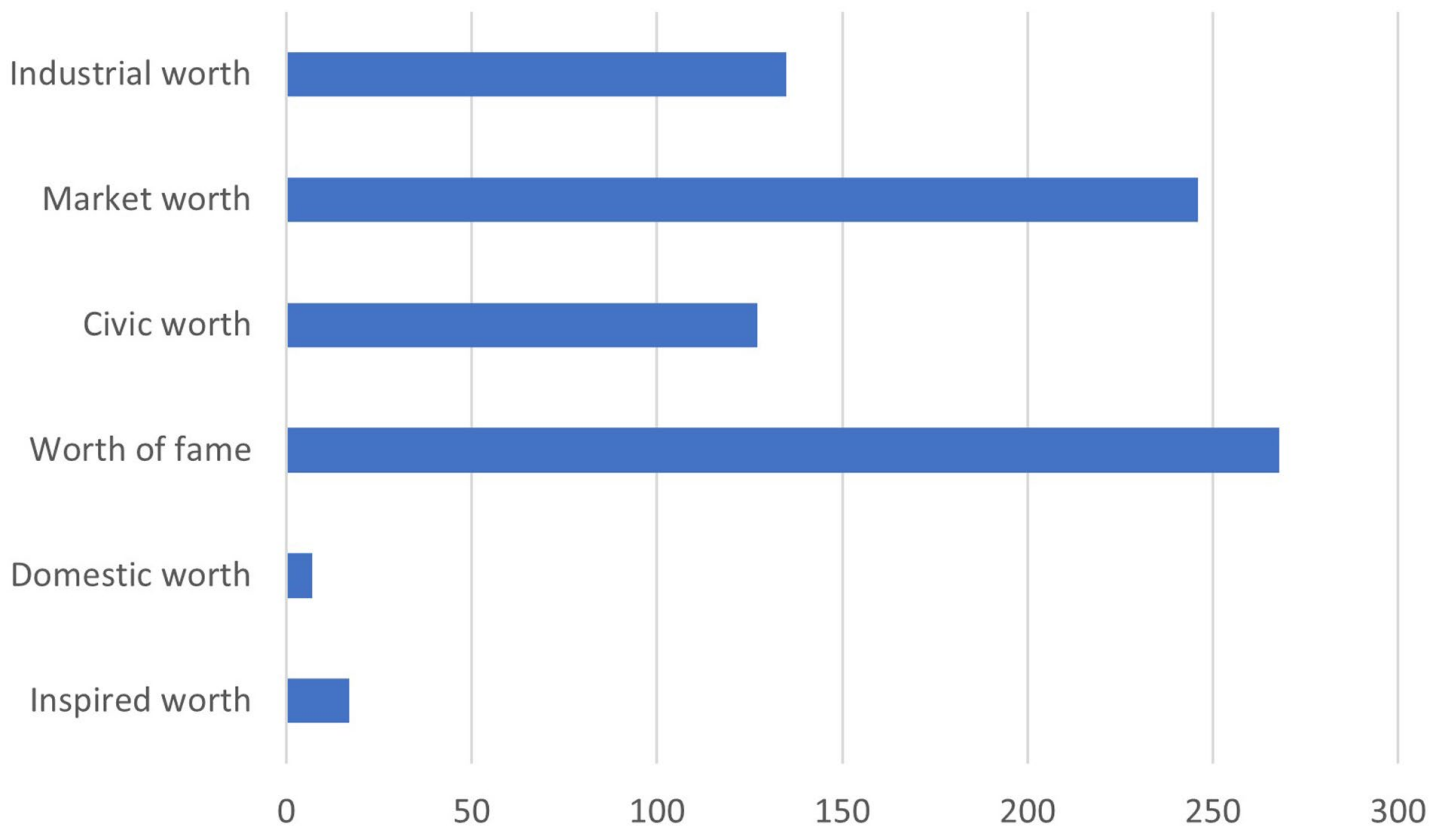
Frequency of different justifications in the exclusion policies of TFFP signatories.

The institutional investors who have signed the Tobacco-Free Finance Pledge discuss a variety of justifications behind their exclusion policies and RI approach in general in their PRI report. These justifications largely correspond to the orders of worth described by Boltanski and Thévenot (51). Figure 1 displays the frequencies of claims using each justification in the data.

The most frequent order of worth among the TFFP signatories was that of fame. Many signatories referred to compliance with international standards and reputational risks to justify their RI approach. This suggests that the de-normalization of a product or sector, but also the change in the public perception of an industry, may be a powerful incentive for investors to implement exclusion policies. Similarly, influential opinion leaders and international norms or conventions can change investor behavior. The fame-related justifications were subdivided into two subcategories: those that pertain to compliance with international norms or recommendations from influential organizations such as the United Nations, and those dealing with mitigating reputational risks:

*Divestment and restriction of investment from shares in tobacco producers as well as controversial weapons is applied across our listed equity portfolio. These exclusions are a commitment by us to uphold good standards of ESG and remove exposure to industries which contravene international conventions as well as to promote increased social standards by addressing key health concerns across the world* (signatory n.144, UNPRI report 2020, p. 74).

*We believe that some industries and practices will simply no longer have a role in society and see participating in these areas as risk to our reputation as a sustainable investor as well as to the financial risk management our portfolio* (signatory n. 148, UNPRI report 2020, p. 32).

Justifications based on market worth were also commonplace. These were often connected to fame-related justifications. Both market and fame-related justifications share a focus on external factors, rather than following internal values. Typically, the market worth concerned risks that could potentially affect financial performance in the future, such as changes in legislation or consumption habits. This type of argument is typical of financial companies that seek to make a profit and that have a fiduciary duty:

*Our research indicates that the tobacco industry has a bleak financial future and is likely to be unsustainable. Tobacco companies face increased regulation and litigation by governments around the world, which impacts consumer demand, sales, and profits. The industry’s ESG profile is inconsistent with our principles. This was the catalyst for examining its financial sustainability* (signatory n.99, UNPRI report 2020, p. 54).

The third most frequently used argument category pertained to industrial worth. Industrial and market worth-based justifications were frequently associated (33 co-occurrences in total). In these statements, the RI approach focused on the use of metrics, ratings, and performance assessments to drive efficient ESG implementation:

*X has funds implementing integration, screening, and thematic ESG incorporation. For integration, ESG metrics have an influence on selection and weighting of securities in our portfolios* (signatory n.61, UNPRI report 2020, p. 48).

The civic worth was the fourth argument category. The relatively sparse use of civic-related justifications (compared with worth of fame and market worth-based arguments) may stem from the need for institutional investors to favor the interests of their clients rather than the public interest. There were nevertheless some examples of civic justification in the data:

*It is a long-term approach that guarantees that today’s choices will not have negative consequences for future generations* (signatory n.96, UNPRI report 2020, p. 44).

The inspired worth was the fifth category. Only a small number of signatories adopt a faith-based RI approach that aligns with religious values. Similarly to the civic worth, the inspired worth focuses on internal values rather than external factors such as a regulatory change or reputational risk:

*Zero exposure to tobacco* etc. *per above but also divesting other exposures that we do not believe is in best interests of our members or aligned to ESG and Christian values* (signatory n.13, UNPRI report 2020, p. 65).

There were only seven statements in the data that related to the domestic worth. This was the sixth and last category. Each mention of domestic worth highlighted the tradition of RI implementation:

*X defined investment beliefs including sustainability considerations over 35 years ago when the foundation was founded. X is a pioneer in RI among Swiss pension funds and the ethical and ecological focus is well known among beneficiaries since the foundation of X* (signatory n.100, UNPRI report 2020, p. 26).

## 4 Discussion

This study has focused on the sector exclusion of addictive industries among Tobacco-Free Finance Pledge signatories and its potential implications in terms of commercial determinants of health. The results have shown a significant degree of heterogeneity. We found that 47 TFFP signatories (29% of all) did not disclose, at the time of the data collection, any publicly accessible exclusion policy. This confirms the trend observed by Acuti et al. (12), whose study showed that a large part of the “addiction companies” in their sample did not publish any sustainability report, which they explained by the willingness to avoid making disclosures that are not mandatory.

Divergence among the signatories was found in terms of their exclusion thresholds of tobacco, with almost 20 percent of the TFFP signatories still investing in tobacco to some degree. We also found differences in terms of exclusion policies regarding the other addictive industries of alcohol, gambling, and recreational cannabis. Typically, tobacco, as well as other addictive industries, were excluded by larger European asset management-type investors. Investors from other geographical contexts and under different types of investment profiles were less likely to exclude addictive industries. The results are supported by prior research (44, 53), which found that exclusion policies across different sectors were most prevalent among European investors. According to one survey, 80% of surveyed investors in Europe had exclusion policies, compared to 65% among Asia Pacific investors and 40% among North American investors (44). Similarly to our study, previous research has also found variance across European contexts (40, 54). Furthermore, and similarly to our results, other research has found that RI and size of the investor are related in a U-shaped curve, with the smallest and largest institutional investors most likely to apply RI policies (40, 55).

Our qualitative analysis of justifications for exclusion policies shows that investors who choose to apply negative screening do so mainly for reasons of public image management (fame). While financial firms do not produce addictive commodities themselves, they are concerned with the public perception of their investments. The second main justification for sector exclusion was related to market risks. Both justifications are intertwined, as controversies can affect financial firms’ reputation and therefore their valuation (56). However, as Boltanski and Chiapello (57) have noted, capitalism comes with its own normative and moral requirements. While aiming to make profit, investors are also under ethical pressures from stakeholders, shareholders, and the public regarding investments in controversial industries, including addictive industries. The moral justifications used by the TFFP signatories for their general approach to RI, or more specifically for their sector exclusion policies, are consistent with the idea advanced by existing research that capitalism is not a-moral: companies or the societal contexts in which they operate are not value-free [see (58, 59)].

The results therefore highlight the conflicting aims of profitability and morality (60). Often, these aims are contradictory. For example, a survey by Morgan Stanley (61) showed that investors perceived investment performance as a major impediment to the adoption of RI. While some investors may prioritize social responsibility over profitability (26), most investors respond to financial risks rather than ethical principles (60). Investment decisions can therefore become an important commercial determinant of health. If profit motives are highlighted, investment can help grow addictive industries and related harms. However, if investors can be persuaded to divest from addictive industries, this can contribute to de-normalization of use. Reputational risks can be a powerful tool to encourage more responsible investment decisions (56, 61, 62). Negative media coverage, stakeholder pressure, pledges such as the TFFP, or international treaties can have a particularly significant impact on corporate investment practices [e.g., (39, 63, 64)]. The greater the reputational risk, the weaker the incentive to continue investing.

The important variance in terms of exclusion policies toward different addictive commodities is probably also resultant of differences in reputational risks. The tobacco industry is considered by many as ‘the worst industry’ while other addictive industries are ‘not as bad’ [(6), p: 11]. Such a categorization was visible in our study: In addition to tobacco, only gambling is excluded by a relatively large share of the signatories (35 percent). Most signatories do not systematically exclude alcohol or cannabis investments from their portfolios. This suggests that there is indeed a hierarchy of addictive industries that may reflect how controversial they are and how established social understanding of their harm production is. Acuti et al. (12) similarly showed a hierarchy among “addiction industries,” with the tobacco industry emphasizing demarketing strategies in their CSR reports much more than gambling and alcohol companies, which they explained by a greater recognition of tobacco-related health harms.

Exclusion of tobacco manufacturing has become increasingly institutionalized following the implementation of the Tobacco-Free Finance Pledge. Research evidence similarly shows that investors have started to avoid the tobacco industry over time [e.g., (56, 65)]. This cannot be solely attributed to declining consumption of tobacco products, as the number of smokers particularly in the Global South continues to grow (56, 66) and the legalization of marijuana in some jurisdictions may represent new market openings for tobacco companies in the Global North (56, 67, 68). Instead, it is likely that reputational risks in the tobacco industry, and investor pressure to comply with selected ESG criteria, explain the declining value of tobacco stocks (56, 65). In the United States, Canada, and Western European countries in particular, the social acceptance of smoking has changed dramatically from being considered a normal yet bad habit in the 1960s to becoming unwanted and deviant behavior in the 2000s (69).

Gambling and alcohol-related investment has been very profitable (43, 44), but growing public health concerns are likely to cause reputational harm to investors in these sectors (3). Particularly in the gambling field, recent policy changes, particularly in Europe, have augmented consumer protection policies, availability restrictions, and limits on industry practices (70). According to a survey by Morgan Stanley in 2022, the number of investment funds excluding gambling increased by 5 % between 2021 and 2022 (53). European and long-only funds are leading this trend, which is believed to result mainly from changing requirements related to the EU Sustainable Finance Disclosure Regulation (SFDR), as no change has been observed in North America or among hedge funds (53). Similarly in our data, the need to avoid reputational risks was often mentioned. The SFDR came into force in 2021 and set stricter transparency obligations on ESG criteria for financial companies operating within the EU, which must give stakeholders clear information on the sustainability of their investments (80). This may partly explain why European institutional investors in our sample implemented more exclusion policies than their counterparts from other regions.

Similar findings were observed by Acuti et al. (12), who found that European-based “addiction companies” tend to report more on their ESG criteria than others, which they believe is likely due to the European Non-Financial Reporting Directive. Additionally, the trend observed in our data is likely to increase. The Corporate Sustainability Reporting Directive (CSRD), a directive entered into force in 2024 to improve the climate and social environment, requires large companies operating in the EU to publicly report on the sustainability of their investments (71). Institutional investors in the EU are therefore likely to face growing pressure to demonstrate their commitment to ESG criteria, especially in controversial sectors such as gambling (53), compared to their counterparts in other regions. However, stricter RI policies in the EU do not prevent the gambling industry from expanding globally.

Like tobacco or alcohol companies before, the gambling industry from the Global North is increasingly targeting markets in the Global South for expansion [e.g., (72)]. In the case of recreational cannabis, the still ongoing legalization in many jurisdictions is only starting to normalize its use (73, 74). The low exclusion levels of recreational cannabis in our study are likely explained by the fact that investors have not yet included it in their negative screening. If development follows a similar path to that of tobacco before, investor interest in the cannabis industry is likely to grow before public health concerns will bring reputational risks.

Investing in addictive sectors contributes to the spread of harmful consumption and related negative consequences on public health globally by allocating capital to operators involved in these sectors. Advocacy and public health campaigns are needed to target regulations on addictive industries but also in investment to these sectors [*cf.* (20)]. Institutional investors have a fiduciary duty to act in the best interests of their beneficiaries. This legal obligation often clashes with an investment strategy that prioritizes ESG criteria. However, and as also argued by Richardson (60), financial self-interest can be a powerful motivator in changing investor behavior. Framing sustainability issues as financial threats or opportunities is therefore more likely to resonate with investors than moral arguments. This type of de-normalization of harmful investments will in turn limit the growth and related harms of these sectors. The Tobacco-Free Finance Pledge is a significant initiative in line with the principles of the WHO Framework Convention on Tobacco Control and exemplifies efforts to address a global health issue at a global scale. The TFFP could serve as a model that could be adopted in other addictive industries.

This study has some limitations. The sample size is relatively small, with only 161 institutional investors included. The data may not be representative of all investors or responsible investors in general. The small size of the sample limits possibilities for multivariate analysis, and the role of recreational cannabis is minor in the sample. Another limitation relates to the fact that the study only investigates sector exclusion based on revenue derived from production, as including revenue from distribution and/or services would have been too restrictive. Likewise, only a few investors apply exclusion thresholds or zero-tolerance policies toward all addictive industries considered. Finally, the sample is cross-sectional; therefore, it cannot capture changes in responsible investment policies toward addictive industries over time. Further research, including comparative and longitudinal studies, would be necessary to fully understand the extent to which investors exclude certain sectors and the relative importance of excluding addictive industries compared to other controversial sectors, such as the arms or the fossil fuels industries.

## 5 Conclusion

This study has shown that there is significant heterogeneity in terms of responsible investment policies toward addictive industries among signatories of the Tobacco-Free Finance Pledge. Most investors do not exclude other addictive sectors. Signatories vary in terms of their exclusion thresholds. The size and geographical origin of the investors are linked to the extent of exclusion practices. Particularly, a European origin was connected to exclusion policies. The analysis of justifications behind exclusion decisions shows that justifications pertaining to compliance with international standards and reputational risks were the most common. This suggests that the de-normalization of a sector may be a powerful incentive for divestment. Investment decisions are an important commercial determinant of health. Investment decisions can contribute to reducing burden of harm, but they can also help grow harmful sectors. International agreements, advocacy, and regulations are needed to encourage ethical investment. The TFFP is unique in that it encourages investors to exclude the tobacco sector. Similar pledges do not yet exist for other addictive industries such as alcohol, gambling, or cannabis but would be recommendable. Divesting from addictive industries is a crucial step toward a public health approach that prioritizes population health over financial profits.

## Data Availability

The raw data supporting the conclusions of this article will be made available by the authors, without undue reservation.
